# Initiative in Work Teams: Lever between Authentic Leadership and Results

**DOI:** 10.3390/ijerph18094947

**Published:** 2021-05-06

**Authors:** Ana Lisbona, Abel Las Hayas, Francisco J. Palací, Michael Frese

**Affiliations:** 1 Departamento de Psicología Social y de las Organizaciones, Universidad Nacional de Educación a Distancia, Juan del Rosal 10, 28040 Madrid, Spain; alashayas2@alumno.uned.es (A.L.H.); fpalaci@psi.uned.es (F.J.P.); 2 Asia School of Business, (In Collaboration with MIT Sloan Management) Sasana Kijang, Kuala Lumpur 50480, Malaysia; Michael.frese@asb.edu.my; 3 Department of Management and Organization, Leuphana University of Lüneburg, 21335 Lüneburg, Germany

**Keywords:** authentic leadership, initiative, work engagement, productivity, teams

## Abstract

*Background*: The central point of this study is *team initiative*, and we analyzed how the theoretical model of antecedents and consequents of personal initiative contribute to explaining the relationship between team initiative and its antecedents and consequents. Authentic leadership is proposed as the antecedent, and the consequent leads to two types of outcomes, one of which is related to employee well-being, and the other is related to performance. However, little is known about what occurs in this relationship once the focus shifts to the team level. From a team perspective, with the label *team initiative*, we propose a collective construct defined similarly to personal initiative. This study shows the relationship between team initiative and its two consequences, team work engagement and performance, which are measured in terms of team productivity by the leader. *Methods*: Our model was tested in a field study with 344 employees of 79 work teams belonging to 55 organizations. *Results*: The analysis of the results using SEM and a regression analysis supported our main hypotheses. *Conclusions*: The finding that initiative is related to performance establishes the importance of initiative at the team level. It also emphasizes its impact on employee well-being through team work engagement and suggests the importance of authentic leadership.

## 1. Introduction

Organizations must address numerous and growing challenges in various fields, including economics, technology, law, society, and environment, in a sustainable and ethical manner in a business setting characterized by complexity, uncertainty, and volatility. Thus, the competitiveness of organizations increasingly requires active performance by their employees. A wide range of behaviors, including flexibility, adaptability, learning, and improvement, is expected of employees beyond the mere fulfilment of their tasks [1].

When we talk about going further in tasks, we are referring to proactive behaviors; if we also think about flexibility and continuous improvement, the key concept is personal initiative. Leaders who will improve effectiveness in this context must also adapt to these challenges, which is why the leadership style that has been considered in this research is authentic leadership.

The objective of this work was to analyze the key role of initiative in work teams as a link between an authentic leadership style and results considering well-being one important outcome, evaluated through team work engagement and productivity.

### 1.1. Personal Initiative and Team Initiative

Personal initiative is a form or label of proactive behavior between the frame of active performance, which emphasizes behavior, and the interaction between personality and environment [2]. Personal initiative is defined as a work-oriented behavioral syndrome and is characterized by being the following: (a) *self-starting* (i.e., pursing self-set goals), (b) *proactive* (i.e., anticipating problems and opportunities), (c) *persistent* (i.e., overcoming barriers), (d) *pro-organization* (i.e., consistent with the goals and mission of the organization), and (e) able to *modify the environment* [3,4,5,6,7,8]. As a broad set of behaviors, personal initiative is influenced by factors in the work environment and organizational variables [9]. At the same time, a consequence is that the environment is modified by the individual, which occurs according to the position of reciprocal determinism that underlies the Social Cognitive Theory [10].

As a form of active performance, individual and organizational performances are the principal personal initiative outcomes according to the theoretical model of antecedents and consequences of personal initiative [3]. The issue is how to assess individual and organizational performance. Empirical research studies have found positive relationships between personal initiative and employment, company profitability, career development and self-employment [3], employee performance [3,11,12], problem-oriented coping [3,13], quality of creative ideas [14], implementation of ideas [15], passion [16], entrepreneurial success in small businesses [17], and innovation [14,15,18]. It has also been shown that team initiative is positively related to productivity [19].

There are other relevant outcomes focused on the employees that possibly affect their health and well-being, but few studies have identified the positive or negative consequences related to employee well-being and employee health.

Some studies have analyzed the costs, or negative consequences of showing proactivity. Based on the assumption that proactive behaviors demand compensatory effort due to limited resources, and because proactivity bears the potential of creating uncertainty and social friction, Fay and Hüttges [20] hypothesized that daily proactivity has detrimental effects on daily well-being, although they found another consequence that could be considered a benefit of proactivity as follows: Evidence shows a significant link between proactivity and work overload that could be a benefit because some proactive actions result in immediate improvements and may make up for lost time.

Proactivity is negatively associated with burnout [21], and there are some studies that consider well-being variables as an antecedent to proactivity (e.g., [12,22]). We proposed that work engagement is a consequence and a measure of psychological well-being related to the labor context, as opposed to burnout.

As success is better guaranteed by team work and there are few jobs where individuals can work on their own, a group perspective is demanded. In addition, work teams have positive outcomes: They may offer opportunities for job enrichment, accommodate the need for autonomy of workers, and decrease the workload of supervisors or increase performance on tasks that are too complex for individuals [23]). At its best, teamwork offers a way of synthesizing individuals’ knowledge, skills, and abilities to achieve exceptional creativity, innovation, and productivity [24].

Although research and practice have shown the key role played by team work in achieving organizational efficiency and competitiveness [25,26], proactive behavior scholarship has focused on the individual level. There are some exceptions, such as the team proactive performance concept [27], research about team initiative [19,28,29], or climate for initiative at the organizational level [11].

Team initiative, a collective construct that is defined similarly to personal initiative [3], is a behavior syndrome that results in a work group taking an active and self-starting approach toward achieving work goals and tasks and persisting in overcoming barriers and setbacks [19]. As a collective phenomenon, team initiative could be considered a shared attribute. Team initiative would have originated in the experiences, attitudes, perceptions, values, cognitions, or behaviors that the team members have in common. Within the context of proactivity or active performance, several authors have proposed engagement as a powerful antecedent of performance in organizations and have implied that it should not only be regarded exclusively as an individual-level construct but also as a team-level phenomenon [24,30,31].

As personal initiative, the most relevant team initiative outcome would be increased performance, in this case at the team level, and therefore we hypothesized the following:

**Hypothesis** **1:**  *Team initiative is positively related to team productivity.*

This is intended to be the first contribution of this work, to analyze how team initiative contributes to improving productivity.

However, productivity should not be the only result to take into account; under the framework of positive psychology, it is also important to focus on the well-being of employees and not only on the absence of discomfort (in the form of burnout or stress or absenteeism, etc.), in this case, continuing at the work-team level with the concept of team work engagement. 

#### 1.1.1. Team Work Engagement

The concepts of work engagement and personal initiative have been studied in separate studies by authors who have minimal contact with one another. At least three different studies have shown positive relationships between work engagement and personal initiative [12,32,33,34]. 

Work engagement is defined as a positive, fulfilling, work-related state of mind characterized by vigor, dedication, and absorption [35]. Team work engagement is a shared, positive, and fulfilling, motivational emergent state of work that is related to well-being, defined similarly to individual level work engagement [36].

Research that relates work engagement and personal initiative considers work engagement an antecedent or modulator/mediator variable [32,34]. However, there are exceptions, such as those from Bernabé, et al. [33], proposing that work engagement is a consequence of personal initiative in university students, and Hakanen, et al. [12], showing the energizing power of job resources and related gain spirals when work engagement enhances personal initiative and impacts work engagement. We proposed that team work engagement is a team initiative consequence and a measure of psychological well-being related to the labor context and that it is opposed to burnout because it is characterized by the presence of positive work-related feelings [37]. We hypothesized the following:

**Hypothesis** **2:**  *Team initiative is positively related to team work engagement.*

The relationship between initiative and work engagement had already been found in previous studies but not at the team level, which constitutes a second innovation in our work.

#### 1.1.2. Authentic Leadership

Thus far, we have discussed team initiative consequents, but it is necessary to recognize the antecedents. An antecedent of personal initiative is leadership [9], whose role on positive organizational outcomes, both individually and collectively, has been studied extensively in the literature [38]. However, what style of leadership will be the most appropriate to encourage personal initiative in a team?

Authentic leadership is among such models and has been defined as “being true to one’s self” [39,40,41,42,43].

As a multidimensional construct, authentic leadership is articulated by the following four components [44]: (a) *self-awareness*—the leader is aware of his values, identity, emotions, goals, and the consequences of his actions on employees; (b) *balanced processing*—the leader objectively analyses facts and data, both external and self-referenced, without distorting, exaggerating, or denying information; (c) *moral perspective*—the behavior of the leader rests on moral and ethical standards rather than possible group, social, or organizational pressure, producing ethical and transparent behaviors; and (d) *relational transparency*—the leader openly shares information, maintaining relationships with employees based on sincerity and honesty.

The behaviors of the authentic leader foster the involvement and participation of group members in decision-making processes and promote information sharing, which, in turn, makes it easier for people to feel more secure and in control of their environment, which increases the proactive behavior of employees [45,46]. A relationship between authentic leadership and proactive behaviors has already been found, so we propose that this relationship also be found with personal initiative and at the group level. There is a significant positive correlation between authentic leadership and the proactive behavior of subordinates [47,48]. Consequently, we propose that authentic leadership also increases team initiative because authentic leadership supports teams to take action and to develop new ideas, as well as to support initiatives that are developed by the team.

Last, a meta-analytic review of authentic and transformational leadership demonstrated authentic leadership dominance over transformational leadership when predicting group or organization performance and organizational citizenship behaviors [49]. For all these reasons, the authentic leadership style was chosen over other styles. We hypothesized the following:

**Hypothesis** **3:**  *Authentic leadership is positively related to team initiative.*

Numerous empirical studies have found that authentic leadership promotes multiple positive attitudes and behaviors that improve employee performance. Studies have shown that authentic leadership is positively related to organizational commitment [50,51,52,53], follower satisfaction with a supervisor [40], work performance [54,55,56], greater follower creativity and individual psychological capital [57], perceived team effectiveness, and followers’ extra effort or higher levels of followers performance [51,52,58]. In addition, authentic leadership is negatively related to burnout [56,59] and with higher work engagement [53].

The objectives of the present study were as follows: 1) to contribute to enriching the understanding of the consequences of proactive behaviors (personal initiative), mainly those related to well-being but also to productivity, considering both employees and organizational outcomes; 2) to explore and go farther than the classic antecedent studied in the previous literature and analyze the role of leadership; and 3) to analyze the antecedents and consequents of proactive behavior in the team context. We argue that authentic leadership is an important antecedent of personal initiative and that authentic leadership is related to productivity through personal initiative and team work engagement, integrating hypotheses 1–3 into a more complex model. Therefore, in this study, we empirically contrasted the mediating role of personal initiative in the relationship between authentic leadership and work engagement and productivity at the team level.

We analyzed the mediating role of team initiative, since other research has linked authentic leadership with an increase in creativity in followers [57] and with organizational citizenship behaviors [49]. Why team initiative? We chose to study the mediating role of team initiative because an authentic leader can foster the involvement and participation of group members and can promote information sharing, which, in turn, makes it easier for people to feel more secure and in control of their environment, which increases initiative [45,46]. We wanted to know if this effect would occur at the group level. The role of the leader should be supporting teams to take action and to develop new ideas, encouraging team members to increase work engagement and achieve better productivity.

However, the relationship between leadership and productivity has not received as much attention compared with other relationships [60]. Recently, new leadership models have been proposed that could better relate to the current setting [61], which would allow a more precise understanding of leadership’s influence on productivity.

We hypothesized the following:

**Hypothesis** **4:**  *Team initiative mediates the relationship between authentic leadership and productivity.*

**Hypothesis** **5:**  *Team initiative mediates the relationship between authentic leadership and work engagement.*

**Figure 1:**
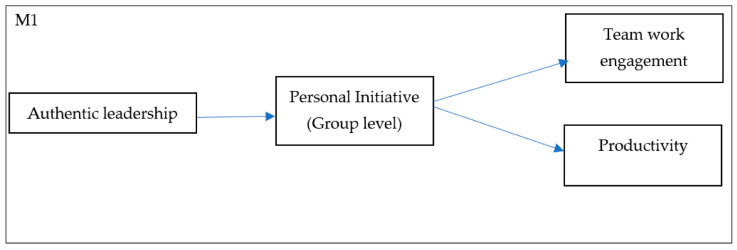
Proposed Model.

These hypotheses are graphically reflected in the model shown in Figure 1.

## 2. Materials and Methods

### 2.1. Samples and Procedure

The sample consisted of 344 employees (51.5% men and 48.5% women) on 79 work teams belonging to 55 organizations. Their mean age was 41.20 years (*SD* = 10.3). They mainly had university (53.9%) and secondary vocational education (10.5%).

Excluding the leader, the 79 work teams were composed of 4 to 8 members (84.3%) or fewer than 4 members (15.6%). They were mainly face-to-face teams (82.4%), and their membership remained stable and continued working as a team.

The organizations were from diverse industry sectors, ranging from consultancy (22.7%), education (19.9%), services (17%), local development (7.2%), health (3.6%), industry (3.6%), logistics (3.6%), public organizations (3.6%), and other sectors (18.8%). The organizations were widely distributed and included multinational companies (49.1%), small family businesses (20%), and medium-sized companies (30.9%).

Organizations were invited to take part in the study, and participation was voluntary. Participants were informed about the objectives of the research, its voluntary nature, and the anonymous and confidential use of the data. Participants completed questionnaires after providing informed consent.

The aim and hypothesis of the study were not disclosed to the contact person in each organization, the leaders, or the team members. The researcher indicated that the aim was to analyze work teams, provided them a work team definition, and explained the inclusion criteria set for teams. The work team definition by Kozlowski y Bell (2003) [25] was explained as follows: (a) two or more individuals who (b) socially interact (face-to-face or, increasingly, virtually); (c) possess one or more common goals; (d) are brought together to perform organizationally relevant tasks; (e) exhibit interdependences with respect to workflow, goals, and outcomes; (f) have different roles and responsibilities; and (g) are embedded together in an encompassing organizational system, with boundaries and linkages to the broader system context and task environment. The following inclusion criteria were set for teams: be active and have minimum activity for 6 months, be composed of a minimum of 3 members (excluding the leader), provide questionnaire replies from at least 2 members per team, have a single team leader who also provides answers to the questionnaire, and have a maximum of 5 different teams per organization.

#### 2.1.1. Operationalization of Variables

To reduce common method biases [62], we obtained measures of the predictor and criterion variables from different sources using the following two questionnaires: one for team members to assess the predictor variables and the other for leaders to assess one of the two criterion variables.

#### 2.1.2. Measures from Team Members

Authentic leadership was assessed by the sixteen items of the ALQ Spanish version [63] (e.g., ‘My leader encourages everyone to speak their mind’; alpha = 0.964). Respondents answered using a 6-point Likert-type scale ranging from 0 (never) to 6 (always).

Team initiative was assessed by seven items [19] (e.g., ‘People in our team usually do more than they are asked to do’; alpha = 0.892). Respondents answered using a 6-point Likert-type scale ranging from 0 (totally disagree) to 5 (totally agree).

Team work engagement was assessed by nine items [64] (e.g., ‘While working, my team feels full of energy’; alpha = 0.925). Respondents answered using a 7-point Likert-type scale ranging from 0 (never) to 6 (always).

Authentic leadership, team initiative, and team work engagement were operationalized by aggregating team members’ scores, following a referent-shift consensus model of composition [65]. Prior to aggregation, we assessed within-team agreement by means of using the average deviation index (AD henceforth) proposed by Burke, Finkelstein, and Dusig (1999) [66]. To interpret the AD values, as Burke and Dunlap (2002) [67] recommended, we used the criterion of AD < c/6, where c is the number of response alternatives used. Authentic leadership and team work engagement were assessed with a 7-point Likert-type scale; thus, we concluded that there was a within-team agreement when the AD values were ≤1.16. Regarding team initiative, a 6-point Likert-type scale was used, and we concluded that there was within-team agreement when the AD values were ≤1. Twelve teams’ AD values were higher than the criterion in one or more variables and were therefore excluded from the analysis. We concluded that the level of within-team agreement in the 79 teams was sufficient to aggregate individual scores at the work team level. The average authentic leadership AD value was 0.66 (SD = 0.28); the average personal initiative (group level) AD value was 0.42 (SD = 0.17); and the average team work engagement AD value was 0.59 (SD = 0.28).

We also computed the intraclass correlation coefficients ICC(1) and ICC(2) [68] because the AD index and the ICCs provide different information. While the AD index is a measure of within-team (or inter-rater) agreement. The ICC(1) is a measure of inter-rater reliability (consistency) that also shows the percentage of variance at the team level. The ICC(2) provides an estimate of the reliability of the team mean [69]. Values higher than 0.12 for ICC(1) and higher than 0.60 for ICC(2) are recommended [68]. The values for ICC(1) and ICC(2) were 0.63 and 0.96, respectively, indicating that the level of consistency of responses among team members and the reliability of the teams’ means on the authentic leadership scale were adequate. The values for ICC(1) and ICC(2) were 0.54 and 0.89, respectively, indicating that the level of consistency of responses among team members and the reliability of the teams’ means on the personal initiative scale were adequate. Additionally, the values for ICC(1) and ICC(2) were 0.58 and 0.91, respectively, indicating that the level of consistency of responses among team members and the reliability of the teams’ means on the team work engagement scale were adequate.

Finally, we carried out a one-way analysis of variance (ANOVA) to determine whether there was a statistically significant between-team relationship discriminating authentic leadership, personal initiative (group level), and team work engagement among the teams. The observed *F* value for authentic leadership was *F*(90, 217) = 2.270, *p* < 0.000. This result shows an adequate between-team discrimination of authentic leadership, and it supports the validity of the aggregated measure [65].

The observed *F* value for personal initiative at the group level was *F*(90, 217) = 1.674, *p* < 0.005. This result shows adequate between-team discrimination of personal initiative at the group level and supports the validity of the aggregated measure.

Finally, the observed *F* value for team work engagement was *F*(90, 217) = 1.561, *p* < 0.001. This result shows an adequate between-team discrimination of team work engagement and supports the validity of the aggregated measure.

#### 2.1.3. Measures from Leaders

Leaders assessed team performance with productivity, the criterion variable. Productivity was assessed by four items [70] (e.g., ‘My team is very efficient in getting maximum output from the resources (money, people, equipment, etc.) we have available’; alpha = 0.726). Respondents answered using a 6-point Likert-type scale ranging from 0 (total disagree) to 5 (total agree).

### 2.2. Data Analyses

First, we performed a confirmatory factor analysis (CFA) using AMOS23 to test a confirmatory factor analysis with three models: (1) a one-factor model where all the constructs were the expression of a single latent factor; (2) a four-factor model where all the factors (authentic leadership, personal initiative, team work engagement, and productivity) were independent; and (3) a four-factor model where all the factors were correlated. If the four-factor model provided a better fit than the one-factor model, it would show that common method variance is less prevalent.

Second, maximum likelihood estimation methods of structural equation modelling (SEM), as implemented by AMOS [71], were used to test the three competing models (see Figure 1). We used various goodness-of-fit indices (absolute and relative indices and parsimony indices). The absolute goodness-of-fit indices were (1) the χ^2^ goodness-of-fit statistic and (2) the root-mean-square error of approximation (RMSEA). The computation of relative goodness-of-fit indices is highly recommended because the χ^2^-test is sensitive to the sample size [72]. Therefore, the *relative* goodness-of-fit indices were calculated with the comparative fit index (CFI) [73]. Additionally, a parsimony and comparative index was calculated with the Akaiké information criterion (AIC) [74]. For the RMSEA, values smaller than 0.08 are considered to indicate an acceptable model fit [75]. For the relative fit index (CFI), values greater than 0.90 are considered to indicate a good fit [76]. For the AIC index, which is an index to compare non-nested competing models, the lower the index is the better the fit of the model.

## 3. Results

### 3.1. Descriptive Analyses

Table 1 presents the means, standard deviations, inter-correlations, and Cronbach’s alphas of all the study variables.

### 3.2. Confirmatory Factor Analysis

We compared the three confirmatory factor analysis models. The fit of the third model with four correlated factors (authentic leadership, personal initiative at the group level, team work engagement, and productivity)( χ^2^ = 1176.055; df = 588; RMSEA = 0.113; CFI = 0.773; AIC = 1404.055) was better than the second model with four independent factors (χ^2^ = 1279.355; df = 594; RMSEA = 0.122; CFI = 0.736; AIC = 1495.355) and the first one-factor model (χ^2^ = 1873.548; df = 594; RMSEA = 0.166; CFI =0.507; AIC = 2089.548).

### 3.3. Model Testing

**Figure 2:**
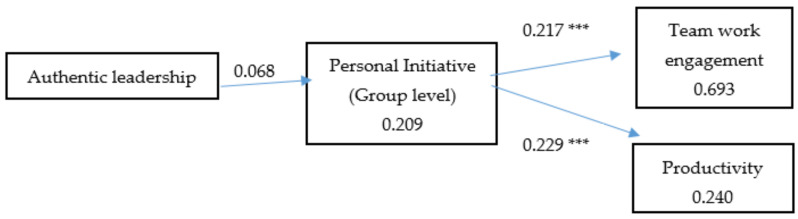
Model 1. Results.

Model 1 (M1) summarized our hypotheses and showed an acceptable fit to the data. All the fit indices were close to their respective criteria (Table 2). This research model was compared with the two alternative models. The fit indices of models M2 and M3 were not as good as those for M1. All the paths of Model 1 were significant (*t* > 1.96) (c.f. Figure 2), but the relationship between authentic leadership with team work engagement and productivity for M2 were not significant.

Personal initiative at group level was related to productivity and team work engagement, and authentic leadership was related to personal initiative. Model 1 explained 20% of the variance in personal initiative, 69% of the variance in productivity, and 24% of the variance in productivity.

The hypothesized mediating role of team initiative between authentic leadership and team work engagement and productivity was tested using PROCESS, an SPSS (IBM, New York, NY, USA) macro created by Hayes (2013) [77] to test conditional process analysis. The macro relies on the re-sampling method of bootstrapping, a procedure that provides an estimate of the indirect effect in the population by resampling the dataset *k* times (5000 iterations in this study) to obtain the indirect effect’s sampling distribution and confidence intervals (CIs). An estimate is considered statistically significant if it is 95% and the CI does not include zero. These findings provided support for Hypotheses 1, 2, and 3.

The first mediation model, with team work engagement as the outcome, showed authentic leadership’s indirect effect on team work engagement through team initiative (group level) (*B* = *0*.226, SE = 0.083, 95% CI = 0.84, 0.41). These findings provide support for Hypothesis 4.

The second mediation model, with productivity as the outcome, showed authentic leadership’s indirect effect on productivity through personal initiative (group level) (*B* = 0.095, SE = 0.0715, 95% CI = 0.002, 0.289), as we proposed in our hypotheses. These findings provide support for Hypotheses 5.

## 4. Discussions

In the current context of change, organizations are deploying different types of strategies to improve their processes and capabilities, and work teams are becoming a central element in the functioning of organizations [25,26]. Similar to other psychosocial variables that thus far have only been studied at the individual level [78], personal initiative is currently being conceptualized at the group level [19]. We found that team initiative is a proactive behavior in the team context and has the following two positive consequences at the team level: work team engagement and productivity.

Individual and organizational performances are the principal personal initiative outcomes according to the theoretical model of antecedents and consequents of personal initiative [1]. But the important thing about our results is that this relationship occurred at the team level and it occurred at the same time as well-being was improved. We must not forget that performance is very important in organizations, but so is well-being and work engagement. Team work engagement is a measure of psychological well-being related to the labor context and opposed to burnout because it is characterized by the presence of positive work-related feelings [37].

Perhaps at the group level we can understand that despite daily proactivity having detrimental effects on daily well-being (Fay and Hüttges (2016)) [20] because there is a link between proactivity and work overload short term, not long term, team initiative may offer opportunities for job enrichment, decrease the workload of the leader or the team members, and even increase performance on tasks that are too complex for individuals [23].

Proactivity is negatively associated with burnout [21], so team initiative could be considered a protector against burnout.

We found support for the relationship between team initiative, or personal initiative at the group level, and team performance, and an evaluation by the supervisor and with the team work engagement. Thus, proactive behavior is understood as a collective phenomenon and is considered a team-shared attribute. It has a positive consequence that involves the whole team, considering that team work engagement is a shared, positive and fulfilling, motivational emergent state of work, relates to well-being, and includes the presence of positive work-related feelings.

The role of the leader, in this case the authentic leader, is very relevant, but the leader does not directly influence productivity and well-being, rather it is the promotion of initiative in the team that achieves productivity and well-being.

### Limitations and Future Research

One limitation of this study is that it was a cross-sectional study; a longitudinal study could demonstrate reciprocal relationships. Nevertheless, in this first study, we observed that leadership relates to team initiative, and that this was related to team work engagement and productivity.

In addition, a longitudinal study would allow us to analyze those possible spirals between initiative and work engagement that have been found by [12], in which work engagement enhances personal initiative and impacts work engagement but not at the group level, spirals that seem very likely due to the inconsistency in previous studies, which sometimes place work engagement as an antecedent and at other times as a consequence or modulator/mediator variable [32,33,34].

We have focused only on the team level, and future efforts should consider multi-level research, including individual and organizational levels.

It is necessary to study more complex models that include more antecedents or environmental supports, among others.

Despite these limitations and that additional work is required, these results contribute to the explanation of the proactive behavior personal initiative, including from the team level perspective, and suggest that team work engagement is the consequence of proactive behaviors and a sign of employee well-being. The results contribute to the explanation of the effect of personal initiative on performance at the team level and establish the basis on which to analyze leadership in the context of active performance.

## 5. Conclusions

Team initiative originated in the belief that the experiences, attitudes, perceptions, values, cognitions, or behaviors that the team members have in common—as well as the leader’s role—would have important consequences. Our results show that authentic leadership is an antecedent of team initiative and that to have an impact on productivity and the team members’ well-being, team initiative is necessary.

To show proactive behavior at the group level, or team initiative, it is necessary that a group take an active and self-starting approach to work goals and tasks and that they persist when confronting and overcoming barriers and setbacks. How a supervisor exercises influence over members is crucial.

Authentic leadership is based on a leader’s authentic character, dedication, and exemplary, ethical, and transparent conduct [63]. This makes it more generic and provides a basis on which other positive aspects of leadership can occur [79]. For example, Rosing, Frese, and Bauschpropose (2011) [79] recommend an ambidexterity theory of leadership to enhance innovation with two complementary sets of behavior; leaders must *reward* or *penalize*, not literally, and foster exploration or exploitation in individuals and teams. The label *ambidexterity* is chosen because it utilizes opening and closing leader behaviors and switches between them to address the ever-changing requirements of the innovation process [80]. The ambidexterity leadership concept is compatible with the generic authentic leadership style.

According to Ilies et al. (2005) [41], leaders influence their followers through five mechanisms: personal and organizational identification; positive emotional contagion, which could be a pre-requisite to achieving a shared, positive and fulfilling, and motivational emergent state of work, such as team work engagement is; positive behavioral modelling; support of self-determination, which is essential to enhance personal or team initiative; and positive social exchanges.

Authentic leaders also share information and provide group members with opportunities to develop collective intuition, expand their knowledge, learn from each other, and acquire new skills. This leads to an increased collective effectiveness (e.g., [52,58,81]), that redound to team proactive behavior or team initiative.

We wanted to develop this approach from positive organizational psychology, which led us to focus on this style of leadership, authentic leadership, and to choose well-being as an outcome measure, considering work engagement rather than considering the negative consequences that burnout can have at the team level.

## Figures and Tables

**Table 1:** Descriptive statistics and correlations for all study variables.

Variables	M	DT	ICC_1_	ICC_2_	AD	1	2	3	4
1. Leadership	4.42	1.12	0.63	0.96	0.66	(0.964)	0.396 **	0.469 **	0.344 **
2. Initiative	4.07	0.68	0.54	0.89	0.42	0.436 **	(0.892)	0.749 **	0.385 **
3. Engagement	4.29	0.95	0.58	0.91	0.59	0.514 **	0.722 **	(0.925)	0.387 **
*3. Productivity*	4.21	0.50				0.074	0.179 **	0.119 *	(0.726)

*Note*. Individual correlations—below the diagonal—(*N* = 310) and team level—over the diagonal—(*N* = 79). Cronbach’s alpha in the diagonal, between brackets; a. Leader measure; b. ** *p* < 0.01; * *p* < 0.05.

**Table 2:** Model fit (*N* = 79).

Models	χ^2^	*Df*	RMSEA	CFI	AIC	Δχ^2^	*df*
M1	976.61	579	0.094	0.847	1222.6		
M2	1077.89	584	0.104	0.810	1313.8	M2−M1 = 101.28 ***	5
M3	1176.05	588	0.113	0.773	1404.1	M3−M1 = 199.44 ***	9

*Note.* RMSEA = root-mean-square error of approximation; CFI = comparative fit index; AIC = Akaike information criterion.

## Data Availability

The dataset used and analyzed during the current study is available from the corresponding author upon request.
